# Genomic analysis highlights the conservation significance of *Torenia concolor* (Linderniaceae) from the periphery of its distribution range

**DOI:** 10.1007/s10265-025-01659-z

**Published:** 2025-08-19

**Authors:** Yuji Isagi, Taiga Shimizu, Yukihiro Kobayashi, Yoshihisa Suyama, Chinatsu Tokuhiro, Goro Kokubugata, Takuro Ito, Kuo-Fang Chung, Atsushi Abe, Takashi Makino, Michimasa Yamasaki

**Affiliations:** 1 Graduate School of Agriculture, Kyoto University, Kitashirakawa-Oiwake, Sakyo, Kyoto 606-8502 Japan; 2 Graduate School of Agricultural Science, Tohoku University, Sendai, Japan; 3 Department of Botany, National Museum of Nature and Science, Tsukuba, Japan; 4 Tohoku University Botanical Gardens, Tohoku University, Sendai, Japan; 5 Biodiversity Research Center, Academia Sinica, Taipei, Taiwan; 6 Botanical Laboratory, Okinawa Churashima Foundation Research Institute, Motobu, Japan; 7 Graduate School of Life Science, Tohoku University, Sendai, Japan

**Keywords:** Conservation value, Deleterious variation, Demography, Endangered species, Isolated population

## Abstract

Biodiversity status assessments are typically conducted on a regional basis. Consequently, there are numerous species that are rare in one region but ubiquitously present in another country or administrative region. Correctly assessing the conservation status and value of such “endangered species” is essential to achieve better biodiversity conservation through the appropriate and efficient use of socioeconomic resources. A comparative genomic analysis was conducted on *Torenia concolor*, which is widely distributed in Southeast and East Asia, but has a limited population in Japan, specifically on Amami Oshima Island. This population has not yet been established as a conservation priority due to the possibility that it may have originated from cultivated plants. We hypothesized that the population was not due to a human-induced distribution; indeed, our findings indicate that the Amami Oshima population is derived from a natural distribution and is phylogenetically unique, retaining comparable genetic diversity with more abundant populations and exhibiting no increase in deleterious variations in their genome. These findings highlight the unique conservation significance of the Amami Oshima population. Furthermore, the findings suggest that this population, being genetically robust, may be sustainably conserved through minimal intervention strategies, such as maintaining current habitat conditions and monitoring population size, as the accumulation of deleterious mutations is comparable to that of the Taiwanese population. This study highlights the importance of accurate assessment of genomic status and contributes to a broader understanding of conservation strategies for regionally rare species.

**Supplementary Information:**

The online version contains supplementary material available at 10.1007/s10265-025-01659-z.

## Introduction

Although species distributions naturally transcend artificial boundaries, such as national borders and administrative areas, assessments of biodiversity status, including those conducted via the Red List, are often carried out on a regional basis. Consequently, numerous species may be rare in one region, while ubiquitous in another country or administrative area (Shibabayashi et al. 2023). It is therefore of the utmost importance to accurately evaluate the conservation status and value of these “endangered species” to enhance biodiversity conservation by employing appropriate and efficient use of socioeconomic resources.

**Fig. 1:**
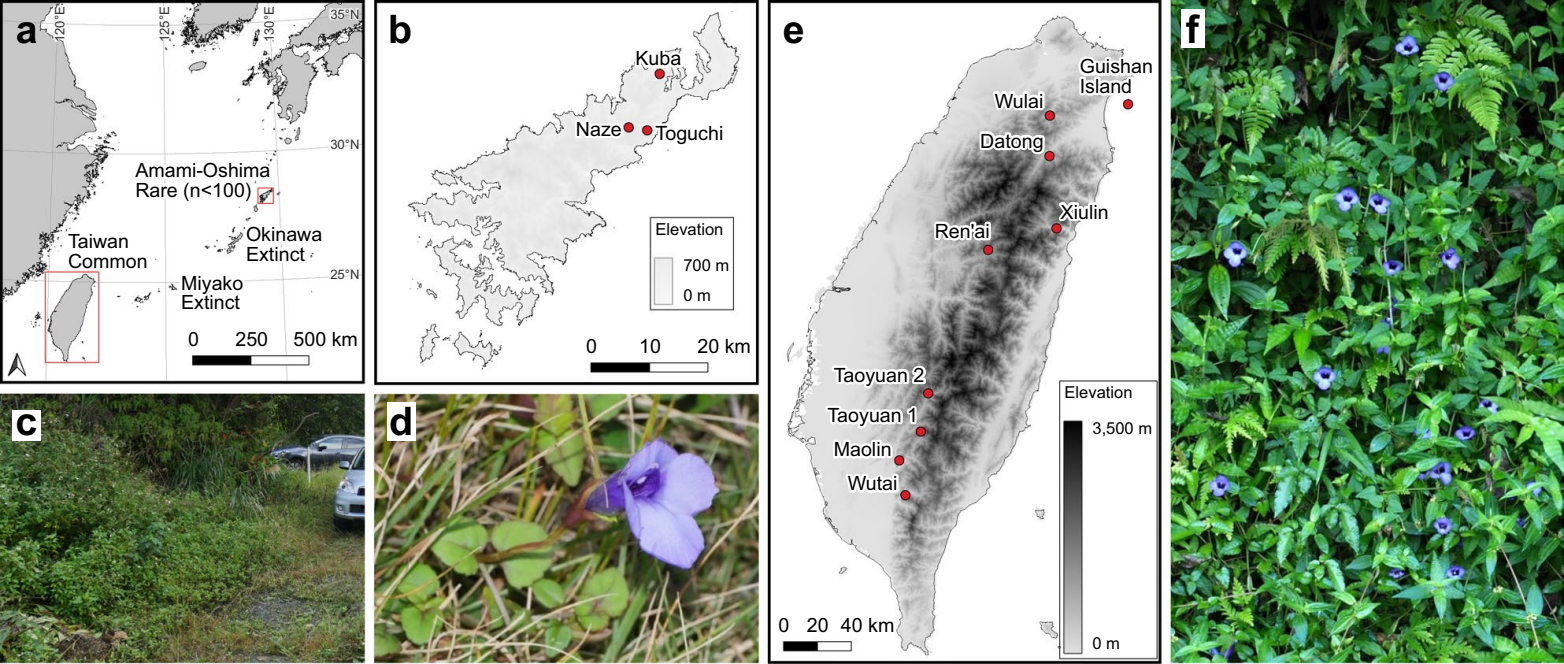
Sampling locations and images of *Torenia concolor*. **a** Sampling sites in Taiwan and Amami-Oshima, Japan. **b** Detailed map of sampling sites in Amami-Oshima. **c** Habitat of *T. concolor* growing along a roadside in Naze, Amami-Oshima. **d** *T. concolor* flowering plant in in Amami-Oshima. **e** Sampling locations in Taiwan. **f** Flowering *T. concolor* plants observed in Yilan County, Taiwan

*Torenia concolor* Lindl., a perennial herb of the family Linderniaceae, is known to thrive in moist grasslands and forest margins across Laos, Vietnam, China, and Taiwan (Ohashi 2017). In Japan, the species is classified as Critically Endangered (CR) and was previously documented on Miyako Island, Okinawa Island, and Amami Oshima Island (Okinawa Prefectural Government 2018). Nevertheless, one population of the species on Okinawa Island has been confirmed extinct, and another is currently of uncertain status. The species has not been recorded on Miyako Island in recent years, and extinction there is presumed to be highly likely (Okinawa Prefectural Government 2018). The extant populations on Amami Oshima Island (Fig. 1a, b) mark the easternmost point of the species’ global distribution range. *T. concolor* has primarily been used, together with other congeneric species such as *Torenia* *fournieri* and *Torenia baillonii* (Laojunta et al. 2019), as a parent in the breeding of horticultural cultivars. Although the original species is rarely distributed commercially, wild populations on Amami Oshima may have originated from cultivated individuals, maintained by enthusiasts of native plants, that escaped and established in the wild. As a result, the presence of some plants, specifically those shown in Fig. 1c and d, in areas adjacent to human settlements has led to uncertainty about the origin of the population, making it difficult to determine whether it should be considered a target for conservation. To address this, it is essential to clarify the origin of *T. concolor* on Amami Oshima and determine its phylogenetic position within the species. This will inform the appropriate conservation value of this species. To this end, we conducted comparative analyses of individuals of this species that grow on Amami Oshima Island and Taiwan (Fig. 1e, f). In comparison to closely related common species, some endangered species have been observed to exhibit a higher accumulation of deleterious mutations which can be associated with vulnerability (Hamabata et al. 2019; Isagi et al. 2020; Kobayashi et al. 2024; Tsunenari et al. 2024). Therefore, we also compared the amount of the accumulated deleterious variations between individuals from common (Taiwan) and isolated (Amami Oshima) populations using transcriptome analysis.

We hypothesize that the *T. concolor* found in Japan, is not a result of human introduction but rather represents a natural distribution. By analyzing the phylogenetic uniqueness and accumulation of deleterious mutations in the Amami Oshima population, we aim to elucidate the phylogenetic distinctiveness and conservation challenges of the isolated populations located on the periphery of the species distribution range.

## Materials and methods

### Plant samples for DNA analysis

We sampled the leaves of a total of 67 individuals of *T. concolor*, including 11 from Tatsugo (Kuba and Toguchi) and 22 from Naze, Amami Oshima Island, 17 from Datong, Yilan County, 1 from Guishan Island, Toucheng Township, Yilan County, 1 from Maolin District, Kaohsiung City, 1 from Ren’ai Township, Nantou County, 2 from Taoyuan District, Kaohsiung City, 1 from Wulai District, New Taipei City, 1 from Wutai District, Pingtung County, and 10 from Xiulin Township, Hualien County, Taiwan, and stored them with silica gel (Table S1). Voucher specimens were deposited at the Herbarium of Tohoku University (TUS; T. Ito 5926, T. Ito 5939, T. Ito 6349, and T. Ito 6355) and the National Museum of Nature and Science (TNS; G. Kokubugata 8882, G. Kokubugata 8890, G. Kokubugata 10033, G. Kokubugata 10349, G. Kokubugata 10787, G. Kokubugata 10861, G. Kokubugata 11090, G. Kokubugata 11091, G. Kokubugata 11095, and G. Kokubugata 11577). We used 67 samples for ddRAD-seq (Peterson et al. 2012), 5 for RNA-seq analysis. In order to perform de novo genome sequencing for PSMC (Li and Durbin 2011), we used 1 sample for MinION long read analysis and 3 for Illumina short read analysis (Table S1).

### DNA extraction and SNPs calling

DNA was extracted from approximately 100 mg of leaf samples using the method of Doyle and Doyle (1987), and the extracted DNA was sequenced using the ddRADseq method (Peterson et al. 2012). The library was prepared according to the protocol described by Sakaguchi et al. (2015). Sequencing was carried out using HiSeq 2500 (Illumina, San Diego, CA, USA) and we only used forward reads for further analysis. The resulting reads were preprocessed using Trimmomatic v.0.33 (Bolger et al. 2014). We set the maximum number of mismatched bases allowed to 2, required the number of bases matched as a palindrome between paired-end reads for clipping to be 30, and required adapter sequences to match at least 10 bases in a read. Reads with an average quality score of less than 20 were removed, and bases with a quality score of less than 19 were trimmed from the beginning and end of the reads, respectively. Reads with a length of less than 100 bp were discarded. We further trimmed the reads to a length of 100 bp using the process_shortreads program in Stacks v. 2.55 (Catchen et al. 2013; Rochette et al. 2019). To identify loci and single nucleotide polymorphisms (SNPs), we used the denovo_map.pl process in Stacks v. 2.2 (Catchen et al. 2011) with the following parameters: -m 3 -M 2 -n 2 –min-maf 0.05 –max-obs-het 0.5 –write-single-snp, without using a reference genome.

### Phylogenetic analysis

For maximum likelihood phylogenetic analysis, a vcf file was generated by selecting SNPs that were shared by 80% or more individuals within each population and using only one SNP per locus. We converted vcf files to phylip format using PGDSpider (Lischer and Excoffier 2012) for each dataset containing all sampled individuals and performed additional SNP filtering to exclude the individuals whose minimum proportion of sites present were less than 0.5 using TASSEL (Bradbury et al. 2007). From the resulting phylip files, we used RAxML-NG v1.0.3 (Kozlov et al. 2019) to construct the maximum likelihood phylogenetic trees with 1,000 bootstrap replicates. The best DNA substitution model was estimated using Modeltest-NG v0.1.7 (Darriba et al. 2020) and the GTR+G model was applied to construct the phylogenetic tree. The resulting phylogenetic trees were edited and visualized using FigTree v1.4.4 (Rambaut 2009).

### Spatial genetic structure

To investigate the spatial genetic structure, we used Bayesian clustering (STRUCTURE ver. 2.3.4; Pritchard et al. 2000) and principal coordinate analysis (PCoA). For STRUCTURE, we ran 10 iterations for each K value of 2 to 9, and for each run, we conducted 100,000 iterations with a burn-in of 100,000. We then used STRUCTURE Harvester (Earl and Bridgett 2012) to estimate the optimal K value. PCoA was performed using PLINK 1.9 (Chang et al. 2015) and R 4.3.2 (R Development Core Team 2009).

### Comparative phylogenetic analysis based on chloroplast DNA sequences

In order to compare interpopulation variations between the Taiwan and Amami Oshima samples with interspecies variations, and to determine the course of intraspecific differentiation through an inclusive phylogenetic analysis with outgroups, we performed phylogenetic analysis using information from shared chloroplast DNA (cpDNA) sequences obtained by mapping reads with BWA-MEM (Li 2013) from RAD-seq data to the complete *T. concolor* cpDNA sequences in China (GenBank: MK789685.1, 153,853 bps) and a congener *Torenia benthamiana* (GenBank: MK789686.1, Cheng et al. 2019, 153,526 bps). This allowed us to extract the chloroplast DNA sequences contained in their DNA sequences obtained by RAD-seq. The phylogenetic relationship was inferred using the maximum likelihood method by RAxML-NG v1.0.3 (Kozlov et al. 2019) with 1,000 bootstrap replicates, based on the shared cpDNA sequence information between all samples. The GTR+G model was applied to construct the phylogenetic tree.

### Demographic inference using PSMC

The past population dynamics of the Amami Oshima and Taiwan populations were analyzed using the Pairwise Sequential Markovian Coalescent (PSMC) method (Li and Durbin 2011). To perform de novo genome sequencing, we extracted DNA from an individual (YI-1205-9) collected from Tatsugo, Amami Oshima, using NucleoBond HWA DNA (Macherey–Nagel). The extracted DNA was treated with the Short Read Eliminator (Japan Genetics Co., Ltd.) to remove low molecular weight DNA. We performed long-read sequencing using the Oxford Nanopore Technology (ONT) MinION on the extracted genomic DNA. Library preparation and sequencing were conducted following the ONT protocol for Genomic DNA by Ligation (SQK-LSK110). The quality and quantity of the obtained reads were evaluated using seqkit (Shen et al. 2016) and nanoplot (De Coster et al. 2018). Short-read sequencing was performed for genome assembly on DNA extracted from a sample YI-1205-9. Library preparation was performed using the Illumina Nextera DNA Flex library prep kit and paired-end sequencing with a read length of 150 bp was carried out on the Illumina Hiseq X next-generation sequencer. Raw sequencing data were processed using Trimmomatic v. 0.39 (Bolger et al. 2014) to remove primer sequences and to trim all reads to a length of 150 bp. Long-read assembly was performed using NECAT (Chen et al. 2021). The required genome size for NECAT was estimated using KmerGenie-1.7051 (Chikhi and Medvedev 2014) based on the Kmer method. The draft genome obtained by NECAT was error-corrected using short reads with MEDAKA (ONT), followed by error correction using short and long reads with hypo (Kundu et al. 2019). To evaluate the quality of the obtained genome, we performed BUSCO analysis (Simão et al. 2015; Waterhouse et al. 2017) using data from eudicots.

Demographic changes were analyzed using PSMC (Li and Durbin 2011) on the samples from Toguchi (YI-1205-9), Naze (YI-1206-9) of Amami Oshima and Datong, Taiwan (YI-1136-16), which were found to belong to different clades based on phylogenetic analysis using RAD-seq. For the latter two samples, libraries were prepared using the Illumina Nextera DNA Flex library prep kit, and 150 bp paired-end sequencing was performed on the Illumina HiseqX platform by Macrogen Japan. Raw data from HiseqX were processed using Trimmomatic v.0.39 (Bolger et al. 2014) for adapter sequence removal and size filtering, as well as BWA-MEM (Li 2013) and SAMtools (Li 2011) with view, sort, and rmdup options for short-read mapping. SNP calling was performed using the mpileup and call options of BCFtools (Danecek et al. 2021). When detecting SNPs, we set the minimum depth to one-third of the average depth and the maximum depth to twice the average depth to prevent the effects of duplicate locus-derived reads. As no literature explicitly provides the generation time (g) of *T. concolor* for PSMC analysis, we assumed it to be three years. This estimation is based on the horticultural treatment of *T. fournieri* as an annual species, our observation that *T. concolor* took over a year to flower under cultivation, and the likelihood that wild conditions are less optimal than cultivated ones. The mutation rate (μ) for PSMC analysis was set to 6.0 × 10^–9^ based on the value of 5.1–7.1 × 10^–9^ estimated for nuclear DNA in angiosperms (Graur and Li 2000).

### Estimation of the amount of deleterious mutations

Adopting the methodology outlined by Hamabata et al. (2019), we conducted transcriptomic analyses to evaluate the conservation difficulty quantifying the accumulation of deleterious variations within the genome. Total RNA was extracted from two (YI-1143 and YI-1154) and three wild individuals (YI-1136-1, YI-1136-2 and YI1136-16) grown in Amami Oshima and Taiwan, respectively, using an Agilent Plant RNA Isolation Mini kit (Agilent Technologies, Santa Clara, CA, USA), following the manufacturer’s instructions. RNA samples were sequenced on an Illumina NovaSeq 6000 with 100 nucleotide paired-end (PE) reads. Reads with over 10% of bases scoring below Q30 were removed via FASTQ Quality Filter in FASTX-Toolkit (http://hannonlab.cshl.edu/fastx_toolkit/). De novo RNA-seq assembly was performed using Trinity ver. 2.2.0 (Grabherr et al. 2011). For transcripts yielding multiple splicing isoforms, the longest isoform with consistent accession across various clusters and genes was chosen from the Trinity output. To identify heterozygous single nucleotide variants (SNVs), all reads were mapped to the reference constructed by Trinity using BWA version 0.7.13 (Li and Durbin 2009), and SNVs were identified with SAMtools version 1.3 (Li et al. 2009). The number of synonymous heterozygous SNVs, which represent putative neutral variations and are representative of genetic diversity, was calculated per kb for each longest transcript.

We used the Protein Variant Effect Analyzer (PROVEAN) (Choi et al. 2012) and Sorting Intolerant from Tolerant (SIFT) (Kumar et al. 2009) to examine non-synonymous SNVs homologous to plant protein sequences in terms of whether each amino acid variation might affect protein function. The default threshold for PROVEAN score prediction of deleterious variation is equal to or below − 2.5, whereas a score above − 2.5 is predicted to be neutral. However, due to the inability to differentiate between original and derived amino acid variants at heterozygous loci in this study, we classified non-synonymous variations with absolute PROVEAN scores greater than |2.5| as deleterious. We regarded amino acid variations with SIFT prediction scores below 0.05 as deleterious and calculated the ratio of deleterious amino acid variations relative to total non-synonymous SNVs in each estimation. SNVs that could not be evaluated by SIFT were excluded from the calculation. The proportion of deleterious non-synonymous variants was calculated for each gene. Furthermore, we quantified the proportion of nonsense SNVs, indicative of loss-of-function, to total non-synonymous SNVs for each species.

### Data availability

RAD-seq data, RNA-seq data, whole genome resequencing data, and MinION data have been deposited at the NCBI Sequence Read Archive and are publicly available under accession number PRJNA1117523 (BioProject).

## Results

### SNP calling

A total of 134,167,465 reads were obtained from 67 samples using RAD-seq, with an average of 2,002,499 reads per sample for 51 bp paired-end sequencing. After executing the denovo_map.pl processing in Stacks, we identified 9,068 SNPs that were shared by more than 80% of all samples in this dataset. Three samples (YI-1198, YI-1199, and YI-1205-1) were excluded due to high levels of missing data, and the remaining 64 samples were used for subsequent analyses.

### Phylogenetic analysis

**Fig. 2:**
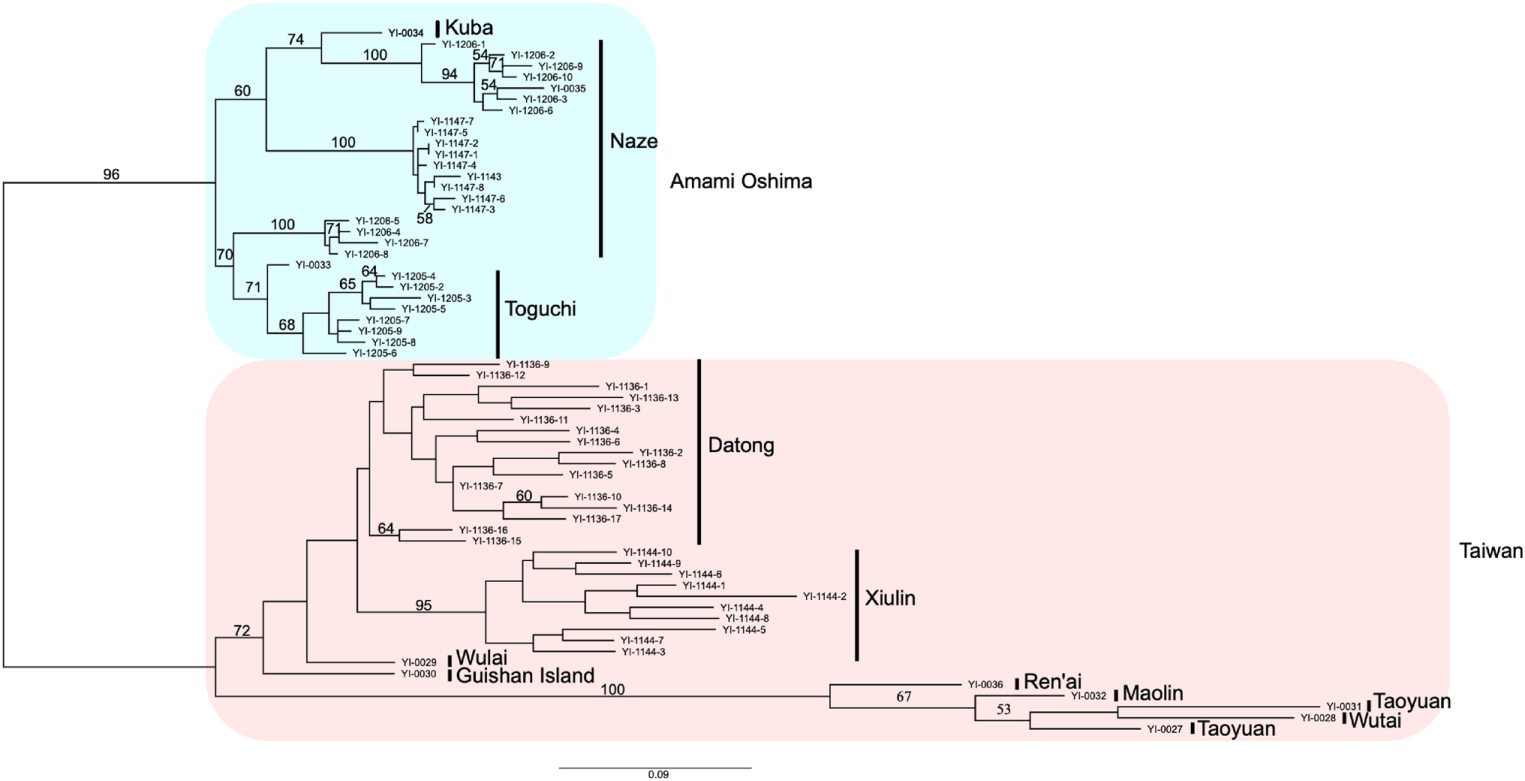
Phylogenetic tree based on RAD-seq SNP data inferred using RAxML-NG. The log likelihood value was −62,886.693284. The numbers on branches were bootstrap values from 1,000 replications. The bootstrap values lower than 50 were not shown

The results of the phylogenetic analysis based on SNPs from RAD-seq revealed two distinct clades corresponding to the Amami Oshima and Taiwan populations (Fig. 2). The Amami Oshima clade and Taiwan clades were clearly distinguished, and several subclades that were associated with the sampling locations were observed. The branch length within the Naze and Toguchi populations of Amami Oshima was comparable to that of the Datong and Xiulin populations of Taiwan (Fig. 2).

### Spatial genetic structure and genetic diversity

**Fig. 3:**
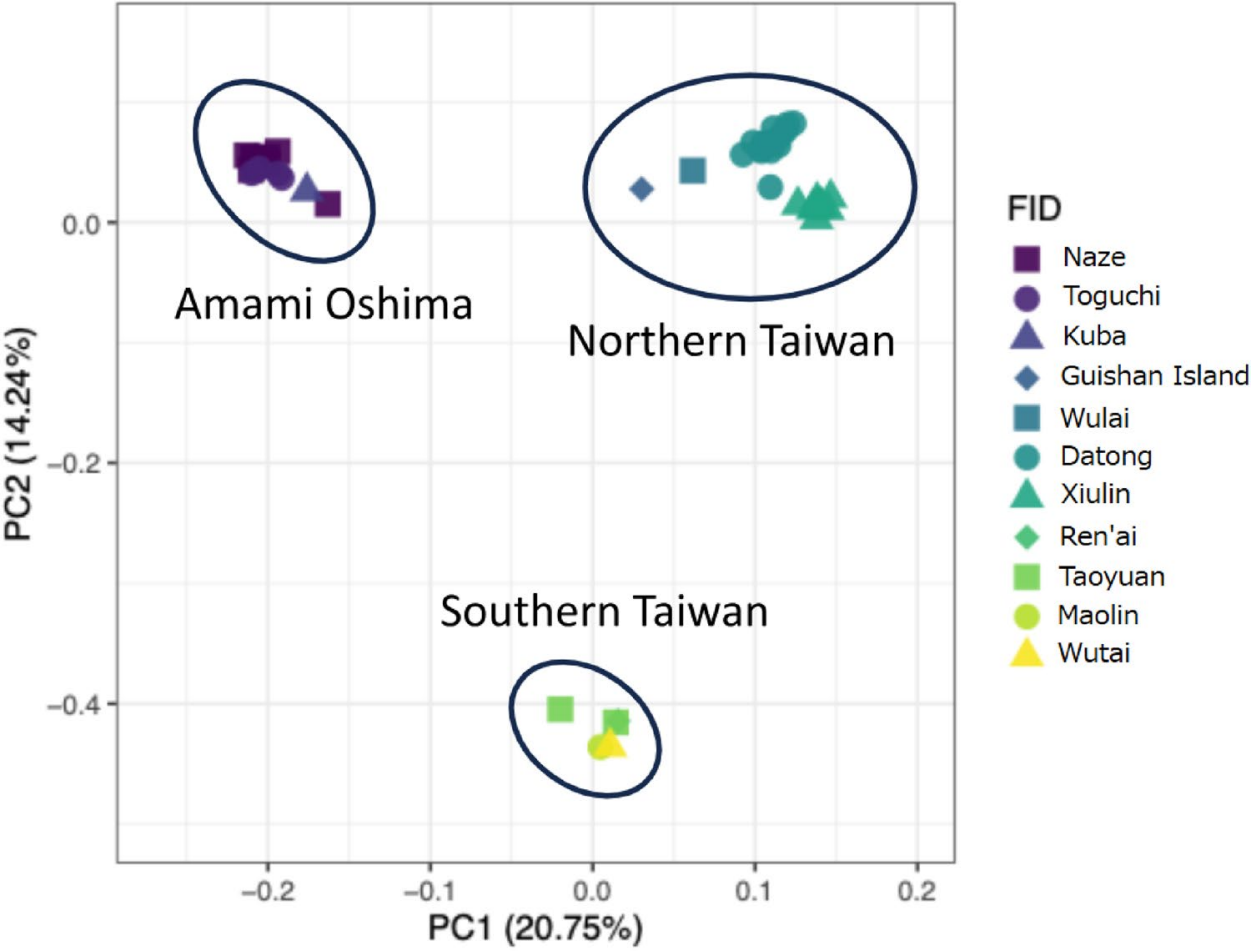
PCoA plot illustrating the population structure of Amami Oshima and Taiwan populations of *T. conclor*

**Fig. 4:**
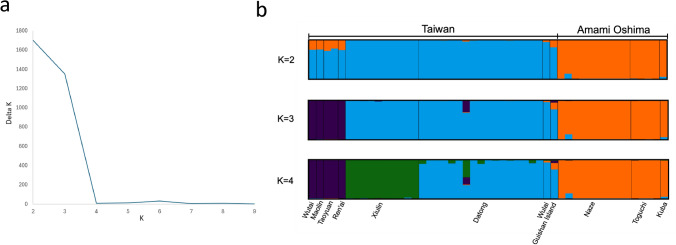
Population structure of Amami Oshima and Taiwan populations estimated by STRUCTURE analysis. **a** Relationship between K and Delta K from STRUCTURE HARVESTER. **b** Population structure estimated by STRUCTURE analysis with K = 2, 3 and 4

A Principal Coordinate Analysis (PCoA) for population structure analysis using PLINK and R revealed that the Amami Oshima populations grouped together as one, while the Taiwan populations divided into two groups, northern and southern Taiwan, on a PC1-PC2 plane (Fig. 3). The result of STRUCTURE HARVESTER indicated that the delta K was highest at K = 2, followed by K = 3, and 4 (Fig. 4a). The Amami Oshima populations and the Taiwan populations were identified as distinct clusters within the results of the STRUCTURE analysis at K = 2 (Fig. 4b). As the K value increased to 3 and 4, the Taiwan populations were divided into 2 and 3, while the Amami Oshima population remained cohesive into one (Fig. 4b). The heterozygosity between the Amami Oshima and Taiwan samples was not significantly different in terms of average and variance (Table 1).

**Table 1:** Average and variance of Ho of Amami Oshima and Taiwan populations

Location	DNA sites	Average Ho	Variance	Mann–Whitney U	Levene’s test
Amami Oshima	Variant site	1.947E−01	3.707E−02	U = 288.0	F = 1.123
Taiwan		1.528E−01	3.458E−03	*p* = 0.747	*p* = 0.294
Amami Oshima	All site	2.775E−04	1.059E−08	U = 257.0	F = 4.410E−05
Taiwan		2.756E−04	1.030E−08	*p* = 0.763	*p* = 0.995

### Comparative phylogenetic analysis based on chloroplast DNA sequences

**Fig. 5:**
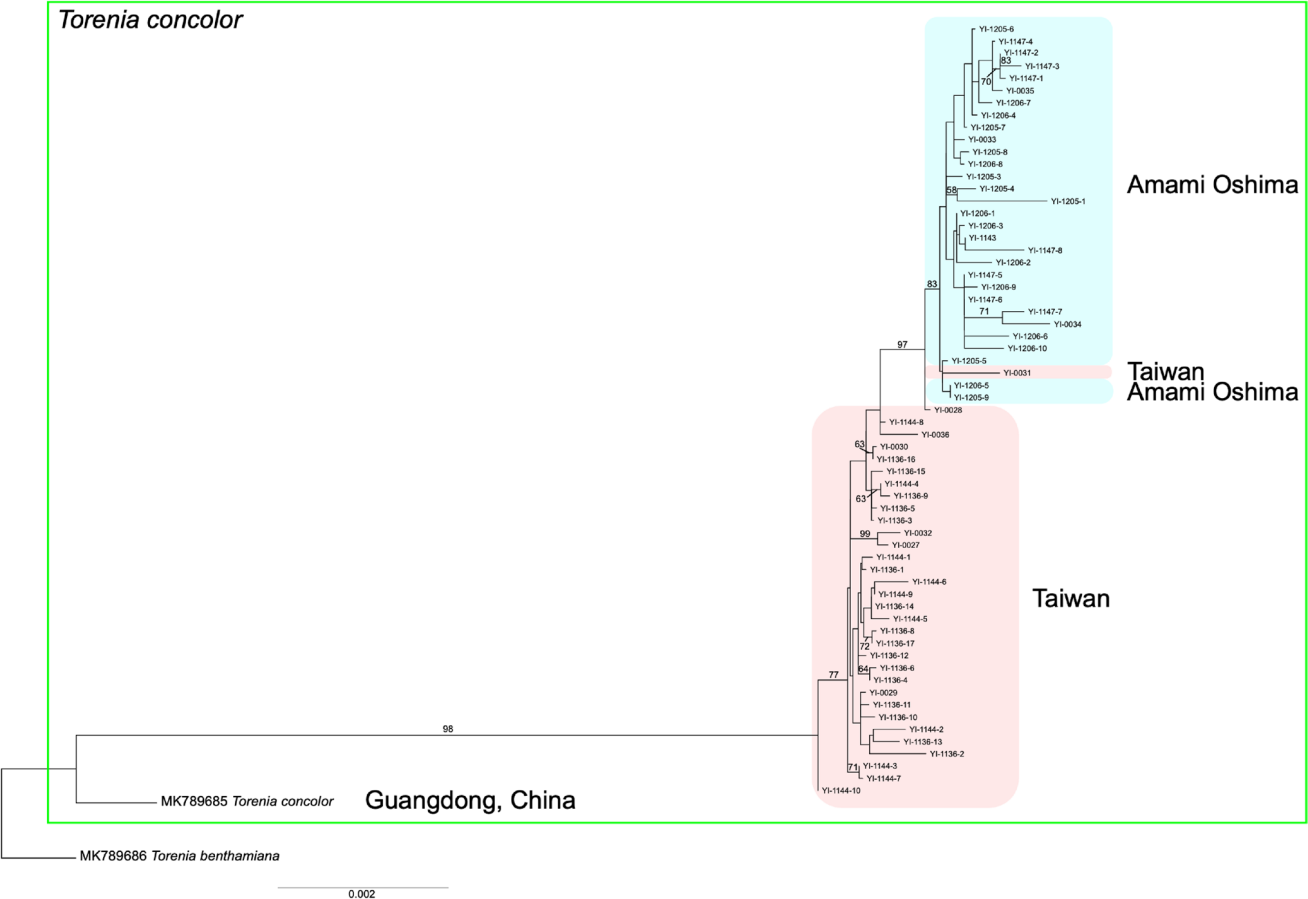
Phylogenetic tree based on chloroplast sequences assembled from RAD-seq short reads inferred using RAxML-NG. The log likelihood value was −53,637.980483. The numbers on branches were bootstrap values from 1,000 replications. The bootstrap values lower than 50 were not shown

We mapped RAD-seq reads to the complete *T. concolor* chloroplast genome and extracted 52,615 to 74,763 homologous bases per sample (average 62,313 bps) for phylogenetic analysis. The length of the aligned sequence matrix was 76,928 bps and the rate of missing data was 21.8%. The result suggests that *T. concolor* diverged from *T. benthamiana* and subsequently expanded from Guangdong Province, China, to Taiwan and the Amami Oshima Islands (Fig. 5). While chloroplast DNA sequences are relatively conservative and exhibit few mutations, it is noteworthy that the branch lengths among the individuals comprising the small Amami Oshima population are comparable to those of the Taiwanese population (Fig. 5).

### De novo* genome sequencing and demographic inference by PSMC*

The Kmer method was employed on Illumina’s short reads with KmerGenie (Chikhi and Medvedev 2014), resulting in an estimated genome size of approximately 367 Mbp. The estimated value was used to assemble the long reads from MinION and the short reads from Illumina using NECAT (Chen et al. 2021). Further correction of errors was carried out using MEDAKA (Kahlke 2021) and hypo, resulting in a draft genome with a total base length of 308.3 Mbp, 2,296 contigs, and an N50 value of 320.7 K. The BUSCO (Simão et al. 2015) score was determined to be 95.2%.

**Fig. 6:**
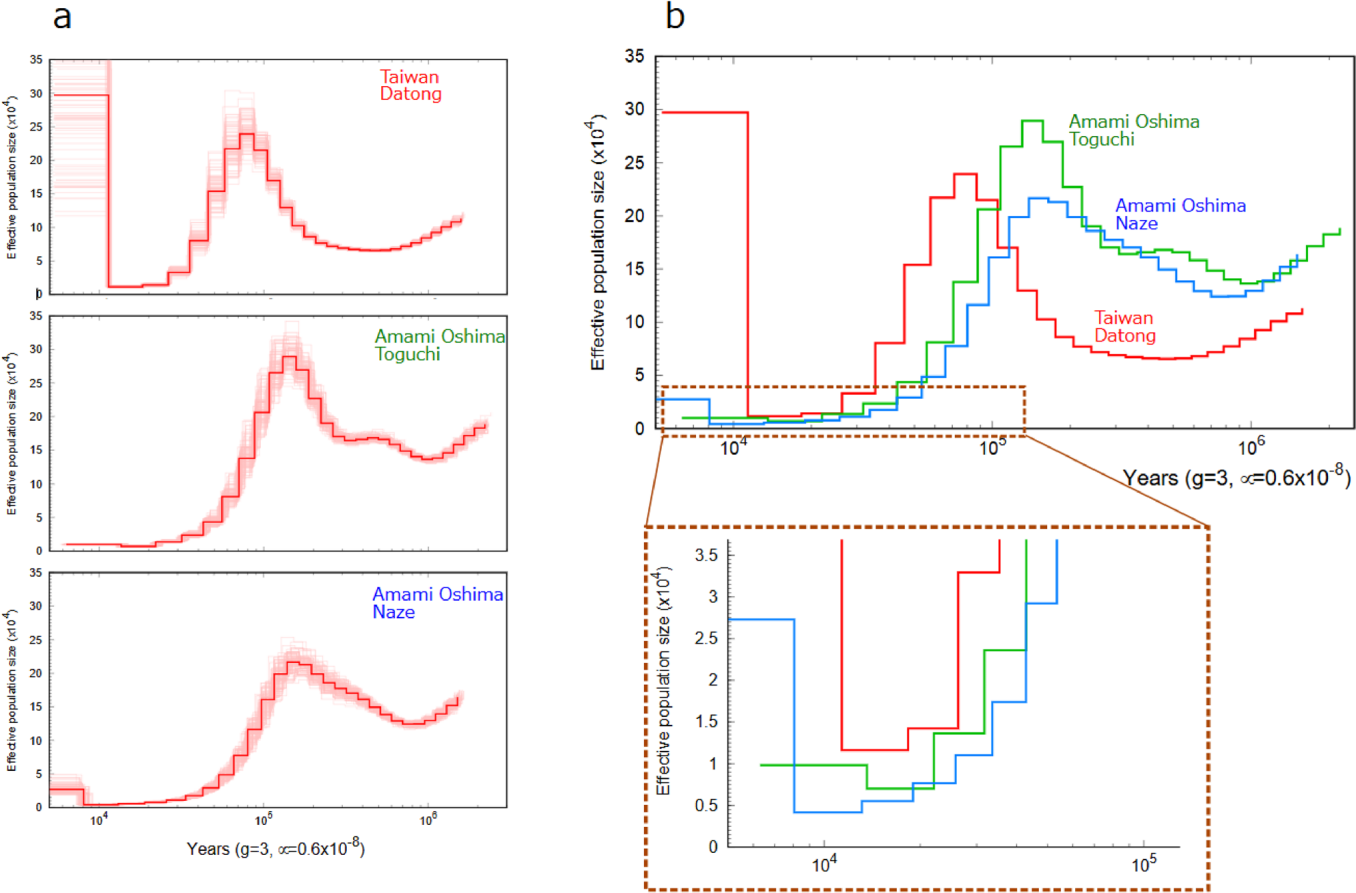
Demographic inference of *Torenia concolor* by using PSMC. **a** The estimated effective population sizes of *T. concolor* from different regions. Thin lines in each figure showed the results of 100 bootstrap replications. **b** The results of the three different regions shown in a single figure

The PSMC analysis facilitated the estimation of the demographic history of effective population size from 4,000 to 10 million years ago. However, given the considerable variance in the estimates, it is deemed that the most recent period for which they are reliable is approximately 10,000 years ago (Fig. 6a). The population size of Amami Oshima exhibited an increase from approximately 400,000 to 100,000 years ago, followed by a precipitous decline. In contrast, the Taiwanese population experienced an increase in population size at a later point than the Amami Oshima population, followed by a similarly rapid decrease. Approximately 10,000 years ago, when the effective population size was most reduced, the effective population size was 4,000—10,000 on Amami Oshima and 12,000 in Taiwan (Fig. 6b).

### Transcriptome analysis

The number of heterozygous synonymous SNVs per kb, which is used as an indicator of genetic diversity, demonstrated no significant difference between the Amami Oshima and Taiwan samples. Furthermore, the values for PROVEAN, SIFT, and nonsense mutations, which indicate deleterious variations, also revealed no significant differences between the two regions (Table 2).

**Table 2:** Genetic diversity and proportion of deleterious variations estimated by transcriptome analysis

Sample	Location			Genetic diversity^a^	Deleterious amino acid variations^b^		Nonsense SNVs^c^
			Non-syn SNVs/total SNVs		PROVEAN	SIFT	
YI-1143	Amami Oshima	Naze	0.3800	0.6675	0.3459	0.2556	0.0067
YI-1154	Amami Oshima	Kuba	0.3841	0.4368	0.3609	0.2803	0.0095
YI-1136-1	Taiwan	Datong	0.3759	0.5618	0.3666	0.2631	0.0087
YI-1136-2	Taiwan	Datong	0.3854	0.6082	0.3441	0.2697	0.0119
YI-1136-16	Taiwan	Datong	0.3940	0.5561	0.3372	0.2586	0.0099
*p* value*				1.0	0.8	1.0	0.4

^a^Genetic diversity based on the number of heterozygous synonymous SNVs per kb on the longest coding sequences

^b^Proportion of deleterious amino variations to homologous to plant protein sequences

^c^Proportion of nonsense SNVs to the total non-synonymous SNVs

^*^Significance analysis of samples from Amami Oshima and Taiwan using the Mann–Whitney U Test

## Discussion

In Japan, *T. concolor* was once prevalent on the main island of Okinawa and Miyako Island. According to the Okinawa Prefectural Government (2018), the species is extinct at one site on Okinawa Island, its status is unknown at another site on the same island, and it is possibly extinct on Miyako Island. Currently, the species is only found on Amami Oshima and the wild population size on Amami Oshima is estimated in the order of tens of individuals at each of Naze, Toguchi and Kuba, and a few individuals at other locations (which were not included in the present analysis), totaling approximately 100 individuals in the wild (Suyama and Kokubugata, personal observation). On Amami Oshima, the plant is typically found in areas subject to human disturbance, such as brightly lit roadsides, suggesting it may have been planted for horticultural purposes and later escaped.

While the present analysis does not encompass samples from the full range of geographic distribution of *T. concolor*, the comparative analysis of chloroplast DNA sequences suggests that after its divergence from a common ancestor with the relatively closely related *T. benthamiana*, *T. concolor* expanded its range across mainland China, Taiwan, and Amami Oshima (Fig. 5). Although comprehensive phylogenetic studies of the genus *Torenia* are still lacking, a recent plastid genome-wide phylogenetic analysis of 28 Linderniaceae taxa, including 11 *Torenia* species (Yan et al. 2023), supports that *T. benthamiana* is relatively closely related to *T. concolor*, following *Torenia asiatica*. The populations from Taiwan and Amami Oshima exhibited a significant phylogenetic distance from those in mainland China (Fig. 5). Moreover, the Amami Oshima populations were evidently disparate from the Taiwanese populations and exhibited diverse lineages within the island (Figs. 2, 5), with genetic diversity equivalent to that of the Taiwanese population (Table 1). In the chloroplast genome analysis, one individual from Taiwan (YI-0031) was found to be nested within the Amami Oshima clade (Fig. 5). This pattern is best explained by chloroplast capture through historical seed-mediated dispersal from Taiwan to Amami Oshima, followed by hybridization with local individuals. Such introgression may have resulted in the retention of the Taiwanese chloroplast haplotype despite assimilation into the nuclear genomic background of the Amami Oshima population. Given that the phylogenetic relationships among the Taiwanese samples (Fig. 2) align closely with their geographic distribution, and no evidence of human-mediated introduction or mixing was detected, it is reasonable to infer that the samples collected and analyzed from Taiwan are derived from naturally occurring populations. Accordingly, the population of this species on Amami Oshima Island is regarded as having particular significance from a conservation perspective. In addition to that, each population on Amami Oshima exhibits clear genetic differentiation, emphasizing the need for suitable conservation measures to maintain genetic diversity, phylogenetic uniqueness, and genetic differentiation among populations. Ongoing monitoring of genetic and population dynamics is vital to ensure the success of these conservation efforts.

Demographic analysis using the PSMC method revealed a sharp reduction in the number of individuals in both Amami Oshima and Taiwan tens of thousands of years ago, likely due to the last Ice Age (70,000 to 10,000 years ago). During the period of greatest reduction, the effective population size was approximately 12,000 in Taiwan and 4,000—10,000 on Amami Oshima. This may be attributed to the larger land area of Taiwan compared to Amami Oshima, as well as its more complex topography, which likely provided numerous refugia. Additionally, Taiwan’s more southern location may have allowed it to retain a more favorable habitat compared to Amami Oshima.

Given that actual population sizes are typically much larger than effective population sizes, it is probable that a considerable number of individuals were present in both regions even during the glacial period. As *T. concolor* primarily distributed in subtropical regions (POWO 2023), it is probable that the species experienced a population increase during the current interglacial period. However, the present population on Amami Oshima is much smaller: about 100 individuals, likely due to human-induced habitat destruction and natural vegetational succession, which have reduced the availability of its preferred habitats, namely moist grasslands and open woodlands. Indeed, the population residing predominantly in the island’s lowlands, are susceptible to anthropogenic disturbances. Therefore, protecting these areas from human-induced disturbances and carefully managing vegetation succession are crucial, as the species thrives in well-lit environments.

In small, isolated populations, a higher accumulation of deleterious mutations and inherent genetic vulnerability can occur, as demonstrated by Hamabata et al. (2019) and Isagi et al. (2020) in endemic endangered species of the Ogasawara Islands. In our study, however, no significant difference was found in the amount of deleterious variation between the Amami Oshima and Taiwanese populations. The accumulation of deleterious mutations in small populations is thought to result from reduced effectiveness of purifying selection due to strong genetic drift. While the Ogasawara Islands are typical oceanic islands, where species tend to persist as small, isolated populations, the situation in the Ryukyu Archipelago, to which Amami Oshima belongs, appears to reflect a different geological background. The geological history of these islands offers a plausible explanation for this difference. Based on Osozawa et al. (2012), the Ryukyu Islands became geologically isolated from the Asian continent around 1.55 million years ago but were intermittently reconnected during glacial periods as sea levels fell. Such episodic connectivity may have allowed larger and stable populations to persist. As a result, the accumulation of deleterious mutations in the Amami Oshima population may have been limited compared to that in the more chronically isolated Ogasawara Islands. Such a genomic profile is likely advantageous for the future conservation of the Amami population, implying that relatively simple conservation approaches may suffice to ensure population persistence. Furthermore, its ability to reproduce through both seed and cutting propagation is advantageous for conservation efforts. Despite its small population size, this species is well-adapted to thrive in bright, moist forest margins. Its robust growth characteristics indicate that the population could be sustained through habitat management strategies, such as selectively clearing underbrush and shrubs to maintain open habitat conditions. However, such interventions should be implemented cautiously to avoid unintended impacts on coexisting species. Nonetheless, the critically low population size highlights the need for urgent and targeted conservation measures.

As above, comparative genomic analyses facilitate the assessment of conservation challenges and phylogenetic uniqueness in regionally rare species. This approach enables the efficient allocation of limited socioeconomic resources, supporting the development of appropriate and effective conservation strategies.

## Supplementary Information

Below is the link to the electronic supplementary material.

Supplementary file1 (PDF 127 KB)
