# Supplementary material for: Genomic analysis highlights the conservation significance of Torenia concolor (Linderniaceae) from the periphery of its distribution range
Source: J Plant Res. 2025 Aug 19;138(6):959–69. doi: 10.1007/s10265-025-01659-z (PMC12638373; doi:10.1007/s10265-025-01659-z)
Supplement: Supplementary file 1 — Supplementary file1 (PDF 127 KB) [file 10265_2025_1659_MOESM1_ESM.pdf]

Article title: Comparative genomic analysis has revealed the unique conservation value of a rare plant species *Torenia concolor* (Linderniaceae) growing on the edge of its distribution range.

Journal name: Journal of Plant Research

Authors: Yuji Isagi, Graduate School of Agriculture, Kyoto University; Taiga Shimizu, Graduate School of Agriculture, Kyoto University; Yukihiro Kobayashi, Graduate School of Agriculture, Kyoto University; Yoshihisa Suyama, Graduate School of Agricultural Science, Tohoku University; Chinatsu Tokuhiro, Graduate School of Agricultural Science, Tohoku University; Goro Kokubugata, Department of Botany, National Museum of Nature and Science; Takuro Ito, The Center for Academic Resources and Archives, Tohoku University; Kuo-Fang Chung, Biodiversity Research Center, Academia Sinica, ; Atsushi Abe, Okinawa Churashima Foundation Research Institute; Takashi Makino, Graduate School of Life Science, Tohoku University; Michimasa Yamasaki, Graduate School of Agriculture, Kyoto University.

Correspondence: Yuji Isagi, isagiy@gmail.com

Table S1 Samples of *T. concolor* used for ddRAD-seq, de novo genome sequencing (nano pore and short read), and RNA seq

Analysis	ID	Collection date	Collector	Location	SRA accession
ddRAD-seq	YI-0033, GK11090	25 Dec 2009	Goro Kokubugata	Toguchi, Tatsugo Town, Oshima District, Kagoshima Prefecture	SRR29208524
ddRAD-seq	YI-0034, GK11091	25 Dec 2009	Goro Kokubugata	Kuba, Tatsugo Town, Oshima District, Kagoshima Prefecture	SRR29208523
ddRAD-seq	YI-0035, GK11095	25 Dec 2009	Goro Kokubugata	Naze, Amami City, Kagoshima Prefecture	SRR29208512
ddRAD-seq	YI-1143	17 Jun 2019	Takuro Ito	Hirata-cho, Naze, Amami Oshima, Kagoshima Prefecture	SRR29208501
ddRAD-seq	YI-1147-1, TI5939	17 Jun 2019	Takuro Ito	Hirata-cho, Naze, Amami Oshima, Kagoshima Prefecture	SRR29208490
ddRAD-seq	YI-1147-2	17 Jun 2019	Takuro Ito	Hirata-cho, Naze, Amami Oshima, Kagoshima Prefecture	SRR29208479
ddRAD-seq	YI-1147-3	17 Jun 2019	Takuro Ito	Hirata-cho, Naze, Amami Oshima, Kagoshima Prefecture	SRR29208468
ddRAD-seq	YI-1147-4	17 Jun 2019	Takuro Ito	Hirata-cho, Naze, Amami Oshima, Kagoshima Prefecture	SRR29208460
ddRAD-seq	YI-1147-5	17 Jun 2019	Takuro Ito	Hirata-cho, Naze, Amami Oshima, Kagoshima Prefecture	SRR29208459
ddRAD-seq	YI-1147-6	17 Jun 2019	Takuro Ito	Hirata-cho, Naze, Amami Oshima, Kagoshima Prefecture	SRR29208458
ddRAD-seq	YI-1147-7	17 Jun 2019	Takuro Ito	Hirata-cho, Naze, Amami Oshima, Kagoshima Prefecture	SRR29208522
ddRAD-seq	YI-1147-8	17 Jun 2019	Takuro Ito	Hirata-cho, Naze, Amami Oshima, Kagoshima Prefecture	SRR29208521
ddRAD-seq	YI-1198	14 Jan 2016	Atsushi Abe	Toguchi, Tatsugo Town, Oshima District, Kagoshima Prefecture	SRR29208520
ddRAD-seq	YI-1199	14 Jan 2016	Atsushi Abe	Toguchi, Tatsugo Town, Oshima District, Kagoshima Prefecture	SRR29208519
ddRAD-seq	YI-1205-1, TI6349	27 Aug 2019	Takuro Ito	Toguchi, Tatsugo Town, Oshima District, Kagoshima Prefecture	SRR29208518
ddRAD-seq	YI-1205-2	27 Aug 2019	Takuro Ito	Toguchi, Tatsugo Town, Oshima District, Kagoshima Prefecture	SRR29208517
ddRAD-seq	YI-1205-3	27 Aug 2019	Takuro Ito	Toguchi, Tatsugo Town, Oshima District, Kagoshima Prefecture	SRR29208516
ddRAD-seq	YI-1205-4	27 Aug 2019	Takuro Ito	Toguchi, Tatsugo Town, Oshima District, Kagoshima Prefecture	SRR29208515
ddRAD-seq	YI-1205-5	27 Aug 2019	Takuro Ito	Toguchi, Tatsugo Town, Oshima District, Kagoshima Prefecture	SRR29208514
ddRAD-seq	YI-1205-6	27 Aug 2019	Takuro Ito	Toguchi, Tatsugo Town, Oshima District, Kagoshima Prefecture	SRR29208513
ddRAD-seq	YI-1205-7	27 Aug 2019	Takuro Ito	Toguchi, Tatsugo Town, Oshima District, Kagoshima Prefecture	SRR29208511
ddRAD-seq	YI-1205-8	27 Aug 2019	Takuro Ito	Toguchi, Tatsugo Town, Oshima District, Kagoshima Prefecture	SRR29208510
ddRAD-seq	YI-1205-9	27 Aug 2019	Takuro Ito	Toguchi, Tatsugo Town, Oshima District, Kagoshima Prefecture	SRR29208509
ddRAD-seq	YI-1206-1, TI6355	27 Aug 2019	Takuro Ito	Naze, Amami City, Kagoshima Prefecture	SRR29208508
ddRAD-seq	YI-1206-2	27 Aug 2019	Takuro Ito	Naze, Amami City, Kagoshima Prefecture	SRR29208507
ddRAD-seq	YI-1206-3	27 Aug 2019	Takuro Ito	Naze, Amami City, Kagoshima Prefecture	SRR29208506
ddRAD-seq	YI-1206-4	27 Aug 2019	Takuro Ito	Naze, Amami City, Kagoshima Prefecture	SRR29208505
ddRAD-seq	YI-1206-5	27 Aug 2019	Takuro Ito	Naze, Amami City, Kagoshima Prefecture	SRR29208504
ddRAD-seq	YI-1206-6	27 Aug 2019	Takuro Ito	Naze, Amami City, Kagoshima Prefecture	SRR29208503
ddRAD-seq	YI-1206-7	27 Aug 2019	Takuro Ito	Naze, Amami City, Kagoshima Prefecture	SRR29208502
ddRAD-seq	YI-1206-8	27 Aug 2019	Takuro Ito	Naze, Amami City, Kagoshima Prefecture	SRR29208500
ddRAD-seq	YI-1206-9	27 Aug 2019	Takuro Ito	Naze, Amami City, Kagoshima Prefecture	SRR29208499
ddRAD-seq	YI-1206-10	27 Aug 2019	Takuro Ito	Naze, Amami City, Kagoshima Prefecture	SRR29208498
ddRAD-seq	YI-0027, GK8882	25 Dec 2009	Goro Kokubugata	Taoyuan District, Kaohsiung City, Taiwan	SRR29208497
ddRAD-seq	YI-0028, GK8890	25 Dec 2009	Goro Kokubugata	Wutai District, Pingtung County, Taiwan	SRR29208496
ddRAD-seq	YI-0029, GK10033	25 Dec 2009	Goro Kokubugata	Wulai District, New Taipei City, Taiwan	SRR29208495
ddRAD-seq	YI-0030, GK10349	25 Dec 2009	Goro Kokubugata	Guishan Island, Toucheng Township, Yilan County, Taiwan	SRR29208494
ddRAD-seq	YI-0031, GK10787	25 Dec 2009	Goro Kokubugata	Taoyuan District, Kaohsiung City, Taiwan	SRR29208493
ddRAD-seq	YI-0032, GK10861	25 Dec 2009	Goro Kokubugata	Maolin District, Kaohsiung City, Taiwan	SRR29208492
ddRAD-seq	YI-0036, GK11577	25 Dec 2009	Goro Kokubugata	Ren'ai Township, Nantou County, Taiwan	SRR29208491
ddRAD-seq	YI-1136-1, TI5895	07 Jun 2019	Yuji Isagi	Datong Township, Yilan County, Taiwan	SRR29208489
ddRAD-seq	YI-1136-2	07 Jun 2019	Yuji Isagi	Datong Township, Yilan County, Taiwan	SRR29208488
ddRAD-seq	YI-1136-3	07 Jun 2019	Yuji Isagi	Datong Township, Yilan County, Taiwan	SRR29208487
ddRAD-seq	YI-1136-4	07 Jun 2019	Yuji Isagi	Datong Township, Yilan County, Taiwan	SRR29208486
ddRAD-seq	YI-1136-5	07 Jun 2019	Yuji Isagi	Datong Township, Yilan County, Taiwan	SRR29208485
ddRAD-seq	YI-1136-6	07 Jun 2019	Yuji Isagi	Datong Township, Yilan County, Taiwan	SRR29208484
ddRAD-seq	YI-1136-7	07 Jun 2019	Yuji Isagi	Datong Township, Yilan County, Taiwan	SRR29208483
ddRAD-seq	YI-1136-8	07 Jun 2019	Yuji Isagi	Datong Township, Yilan County, Taiwan	SRR29208482
ddRAD-seq	YI-1136-9	07 Jun 2019	Yuji Isagi	Datong Township, Yilan County, Taiwan	SRR29208481
ddRAD-seq	YI-1136-10	07 Jun 2019	Yuji Isagi	Datong Township, Yilan County, Taiwan	SRR29208480
ddRAD-seq	YI-1136-11	07 Jun 2019	Yuji Isagi	Datong Township, Yilan County, Taiwan	SRR29208478
ddRAD-seq	YI-1136-12	07 Jun 2019	Yuji Isagi	Datong Township, Yilan County, Taiwan	SRR29208477
ddRAD-seq	YI-1136-13	07 Jun 2019	Yuji Isagi	Datong Township, Yilan County, Taiwan	SRR29208476
ddRAD-seq	YI-1136-14	07 Jun 2019	Yuji Isagi	Datong Township, Yilan County, Taiwan	SRR29208475
ddRAD-seq	YI-1136-15	07 Jun 2019	Yuji Isagi	Datong Township, Yilan County, Taiwan	SRR29208474
ddRAD-seq	YI-1136-16	07 Jun 2019	Yuji Isagi	Datong Township, Yilan County, Taiwan	SRR29208473
ddRAD-seq	YI-1136-17	07 Jun 2019	Yuji Isagi	Datong Township, Yilan County, Taiwan	SRR29208472
ddRAD-seq	YI-1144-1, TI5926	12 Jun 2019	Takuro Ito	Xiulin Township, Hualien County, Taiwan	SRR29208471
ddRAD-seq	YI-1144-2	12 Jun 2019	Takuro Ito	Xiulin Township, Hualien County, Taiwan	SRR29208470
ddRAD-seq	YI-1144-3	12 Jun 2019	Takuro Ito	Xiulin Township, Hualien County, Taiwan	SRR29208469
ddRAD-seq	YI-1144-4	12 Jun 2019	Takuro Ito	Xiulin Township, Hualien County, Taiwan	SRR29208467
ddRAD-seq	YI-1144-5	12 Jun 2019	Takuro Ito	Xiulin Township, Hualien County, Taiwan	SRR29208466
ddRAD-seq	YI-1144-6	12 Jun 2019	Takuro Ito	Xiulin Township, Hualien County, Taiwan	SRR29208465
ddRAD-seq	YI-1144-7	12 Jun 2019	Takuro Ito	Xiulin Township, Hualien County, Taiwan	SRR29208464
ddRAD-seq	YI-1144-8	12 Jun 2019	Takuro Ito	Xiulin Township, Hualien County, Taiwan	SRR29208463
ddRAD-seq	YI-1144-9	12 Jun 2019	Takuro Ito	Xiulin Township, Hualien County, Taiwan	SRR29208462
ddRAD-seq	YI-1144-10	12 Jun 2019	Takuro Ito	Xiulin Township, Hualien County, Taiwan	SRR29208461
RNA-seq	YI-1143, TI5939	17 Jun 2019	Takuro Ito	Naze, Amami City, Kagoshima Prefecture	SRR29225313
RNA-seq	YI-1154, GK11091	22 Jul 2019	Goro Kokubugata	Kuba, Tatsugo Town, Oshima District, Kagoshima Prefecture	SRR29228309
RNA-seq	YI-1136-1, TI5895	07 Jun 2019	Yuji Isagi	Datong Township, Yilan County, Taiwan	SRR29225315
RNA-seq	YI-1136-2	07 Jun 2019	Yuji Isagi	Datong Township, Yilan County, Taiwan	SRR29225314
RNA-seq	YI-1136-16	07 Jun 2019	Yuji Isagi	Datong Township, Yilan County, Taiwan	SRR29225316
MinION	YI-1205-9, TI6349	30 Aug 2019	Takuro Ito	Toguchi, Tatsugo Town, Oshima District, Kagoshima Prefecture	SRR29210522
Short read	YI-1205-9, TI6349	30 Aug 2019	Takuro Ito	Toguchi, Tatsugo Town, Oshima District, Kagoshima Prefecture	SRR29210480
Short read	YI-1206-9, TI6355	30 Aug 2019	Takuro Ito	Naze, Amami City, Kagoshima Prefecture	SRR29210479
Short read	YI-1136-16, TI5895	07 Jun 2019	Yuji Isagi	Datong Township, Yilan County, Taiwan	SRR29210478
